# Complete Genome Sequence of Pseudomonas stutzeri Type Strain SGAir0442, Isolated from Singapore Air Samples

**DOI:** 10.1128/genomeA.00424-18

**Published:** 2018-05-31

**Authors:** Carmon Kee, Ana Carolina M. Junqueira, Akira Uchida, Rikky W. Purbojati, James N. I. Houghton, Caroline Chénard, Anthony Wong, Sandra Kolundžija, Megan E. Clare, Kavita K. Kushwaha, Deepa Panicker, Alexander Putra, Nicolas E. Gaultier, Balakrishnan N. V. Premkrishnan, Cassie E. Heinle, Vineeth Kodengil Vettath, Daniela I. Drautz-Moses, Stephan C. Schuster

**Affiliations:** a Singapore Centre for Environmental Life Sciences Engineering, Nanyang Technological University, Singapore; b Asian School of the Environment, Nanyang Technological University, Singapore

## Abstract

Pseudomonas stutzeri strain SGAir0442 was isolated from tropical air samples collected in Singapore. It is a Gram-negative denitrifying bacterium and an opportunistic human pathogen. Its complete genome consists of one chromosome of 4.52 Mb, containing 4,129 protein-coding genes, 12 rRNA subunits, and 62 tRNAs.

## GENOME ANNOUNCEMENT

Pseudomonas stutzeri is a rod-shaped, single polar flagellated bacterium (1) belonging to the class Gammaproteobacteria. P. stutzeri has been well documented to have been isolated from a wide range of natural environments, such as soil (2, 3), marine water (4), and sediments (3). It is an active denitrifying Gram-negative bacterium and an opportunistic human pathogen with low virulence that causes diseases such as urinary tract infection (5), meningitis (6, 7), and pneumonia (8, 9).

An air sample was collected in Singapore (Global Positioning System [GPS] coordinates of 1.345N, 103.679E) using an Andersen single-stage Impactor (SKC BioStage). Strain SGAir0442 was isolated from trypticase soy agar (TSA) (Becton Dickinson) incubated at 30˚C after impacting airborne microorganisms onto agar. The strain was streaked several times to obtain an axenic culture. The isolated strain was cultured in Luria-Bertani (LB) broth overnight at room temperature prior to DNA extraction. Genomic DNA of the strain was extracted using a Wizard genomic DNA purification kit (Promega) based on the manufacturer’s recommended protocol. A SMRTbell template prep kit 1.0 (Pacific Biosciences) was used to construct a whole-genome shotgun (WGS) library, followed by single-molecule real-time (SMRT) sequencing conducted on a PacBio RS II (Pacific Biosciences) sequencer. Additional WGS libraries were constructed using a TruSeq Nano DNA library preparation kit (Illumina), and 300-bp paired-end short reads were generated on an Illumina MiSeq sequencer.

The PacBio RS II platform provided 52,081 long reads (122.49-fold coverage), while the total number of short reads obtained with the Illumina MiSeq sequencer was 829,385 (110.35-fold coverage). The Hierarchical Genome Assembly Process version 3 (HGAP3) (10), implemented in the PacBio SMRT Analysis algorithm version 2.3.0, was used for *de novo* genome assembly of long reads. The complete processing of the assembly includes preassembly, *de novo* assembly with HGAP3, polishing with Quiver (10), and error correction using Pilon version 1.16 (11) with Illumina reads to improve accuracy. The consensus assembly generated one contig with a chromosome size of 4,524,660 bp with an average G+C content of 64.03%.

The complete genome presented 98.3% identity with the Pseudomonas genus, as predicted by the Phylum-specific Automated Phylogenomic Inference Pipeline (Phyla_AMPHORA) (12). Further taxonomical identification was conducted using the average nucleotide identity (ANI) method with Microbial Species Identifier (MiSI) (13), which revealed a 98.2% similarity with the available reference genome Pseudomonas stutzeri DSM4166 (GenBank assembly accession number GCA_000195105).

Annotation of the genome was performed with NCBI’s Prokaryotic Genome Annotation Pipeline (PGAP) version 4.2 (14). The complete genome was predicted to contain 4,309 genes, of which there were 4,129 protein-coding genes (PCGs), 12 rRNA subunits (5S, 16S, and 23S), 62 tRNAs, and 4 noncoding RNAs. The genome also contains 102 predicted pseudogenes. Functional annotation based on Rapid Annotations using Subsystems Technology (RAST) (15–17) classified 117 genes associated with virulence, disease, and defense. The same number of genes was found to have a role in motility and chemotaxis, and 107 genes were found to be involved in nitrogen metabolism, reflecting the nitrogen-fixing characteristic of this bacterium.

### Accession number(s).

The complete genome sequence of Pseudomonas stutzeri strain SGAir0442 has been deposited in DDBJ/EMBL/GenBank under the accession number CP025149.
