# Correction for Kee et al., “Complete Genome Sequence of Pseudomonas stutzeri Strain SGAir0442, Isolated from Singapore Air Samples”

**DOI:** 10.1128/MRA.00737-19

**Published:** 2019-08-08

**Authors:** Carmon Kee, Ana Carolina M. Junqueira, Akira Uchida, Rikky W. Purbojati, James N. I. Houghton, Caroline Chénard, Anthony Wong, Sandra Kolundžija, Megan E. Clare, Kavita K. Kushwaha, Deepa Panicker, Alexander Putra, Nicolas E. Gaultier, Balakrishnan N. V. Premkrishnan, Cassie E. Heinle, Vineeth Kodengil Vettath, Daniela I. Drautz-Moses, Stephan C. Schuster

**Affiliations:** a Singapore Centre for Environmental Life Sciences Engineering, Nanyang Technological University, Singapore; b Asian School of the Environment, Nanyang Technological University, Singapore

## AUTHOR'S CORRECTION

Volume 6, no. 22, e00424-18, 2018, https://doi.org/10.1128/genomeA.00424-18. Page 1: The title should appear as given in this correction.

